# Effectiveness of Influenza Vaccination in Patients with End-Stage Renal Disease Receiving Hemodialysis: A Population-Based Study

**DOI:** 10.1371/journal.pone.0058317

**Published:** 2013-03-13

**Authors:** I-Kuan Wang, Cheng-Li Lin, Po-Chang Lin, Chih-Chia Liang, Yao-Lung Liu, Chiz-Tzung Chang, Tzung-Hai Yen, Donald E. Morisky, Chiu-Ching Huang, Fung-Chang Sung

**Affiliations:** 1 Graduate Institute of Clinical Medical Science, China Medical University College of Medicine, Taichung, Taiwan; 2 Division of Nephrology, China Medical University Hospital, Taichung, Taiwan; 3 Department of Internal Medicine, China Medical University College of Medicine, Taichung, Taiwan; 4 Management Office for Health Data, China Medical University Hospital, Taichung, Taiwan; 5 Department of Public Health, China Medical University, Taichung, Taiwan; 6 Division of Infection, China Medical University Hospital, Taichung, Taiwan; 7 Division of Nephrology, Chang Gung Memorial Hospital, Taipei and Chang Gung Univeristy College of Medicine, Taoyuan, Taiwan; 8 UCLA Fielding School of Public Health, Los Angeles, California, United States of America; National Taiwan University Hospital, Taiwan

## Abstract

**Background:**

Little is known on the effectiveness of influenza vaccine in ESRD patients. This study compared the incidence of hospitalization, morbidity, and mortality in end-stage renal disease (ESRD) patients undergoing hemodialysis (HD) between cohorts with and without influenza vaccination.

**Methods:**

We used the insurance claims data from 1998 to 2009 in Taiwan to determine the incidence of these events within one year after influenza vaccination in the vaccine (N = 831) and the non-vaccine (N = 3187) cohorts. The vaccine cohort to the non-vaccine cohort incidence rate ratio and hazard ratio (HR) of morbidities and mortality were measured.

**Results:**

The age-specific analysis showed that the elderly in the vaccine cohort had lower hospitalization rate (100.8 vs. 133.9 per 100 person-years), contributing to an overall HR of 0.81 (95% confidence interval (CI) 0.72–0.90). The vaccine cohort also had an adjusted HR of 0.85 [95% CI 0.75–0.96] for heart disease. The corresponding incidence of pneumonia and influenza was 22.4 versus 17.2 per 100 person-years, but with an adjusted HR of 0.80 (95% CI 0.64–1.02). The vaccine cohort had lowered risks than the non-vaccine cohort for intensive care unit (ICU) admission (adjusted HR 0.20, 95% CI 0.12–0.33) and mortality (adjusted HR 0.50, 95% CI 0.41–0.60). The time-dependent Cox model revealed an overall adjusted HR for mortality of 0.30 (95% CI 0.26–0.35) after counting vaccination for multi-years.

**Conclusions:**

ESRD patients with HD receiving the influenza vaccination could have reduced risks of pneumonia/influenza and other morbidities, ICU stay, hospitalization and death, particularly for the elderly.

## Introduction

The influenza virus is a common transmissible human respiratory virus that causes significant morbidity and mortality. In the Unites States, influenza can cause hospitalizations of more than 225,000 patients and 36,000 deaths annually [1], [2]. The morbidity and mortality of influenza are increased in people of old ages, immunocompromised patients, and those with chronic diseases [3]. Patients with end-stage renal disease (ESRD) are also at increased risk of influenza complications [4]. Infection is the second leading cause of mortality and a major cause of morbidity in ESRD patients [5]. In addition, mortality rate related to pulmonary infection is likely 10-fold higher in ESRD patients than in the general population [6]. Immune dysfunction, older age, and comorbid conditions, such as diabetes mellitus, malnutrition, invasive dialysis procedures, disruption of skin and mucosa barriers, and nosocomial transmission, contribute to the high infection risk for ESRD patients [4], [7]. The causes of immune dysfunction in ESRD patients include diminished functions in complement activation, neutrophil, monocyte/macrophage, T-cell, and B-cell [8].

Observation studies have reported that influenza vaccination can reduce the risk of deaths and hospitalizations among elderly and high-risk populations [9], [10]. Thus, annual influenza vaccination for ESRD patients is recommended [11]. However, little is known on the effectiveness of influenza vaccine in ESRD patients. Observation studies showed conflicting results.[12], [13], [14], [15], [16] The present study aims to investigate the effectiveness of influenza vaccination in reducing morbidity and mortality for ESRD patients receiving hemodialysis (HD) using population-based universal insurance data, derived from the Taiwan National Health Insurance (NHI) program.

## Materials and Methods

### Data Sources

The Taiwan NHI was integrated from 13 insurance programs in 1995 in Taiwan. This insurance program has covered approximately 99% of the entire 23.74 million people in Taiwan since 1999 [17]. The Taiwan Bureau of National Health Insurance had authorized the National Health Research Institutes (NHRI) to manage reimbursement claims from hospitals and clinics. A committee at the Bureau of National Health Insurance was responsible to randomly select claims and to check the accuracy of claims. NHRI also had been authorized to established data sets for administrative and research uses. We obtained a sub-dataset with one million of population from NHRI for this study. This subset data is similar to the whole population in the distributions in gender and age. The claims data included information on demographic status, date and source of diagnosis, ambulatory care, outpatient and inpatient treatment, dental services, and physicians providing services. Claims data were randomly audited by the insurance system. Data files are linked with scrambled patient identification number to protect the privacy of the patients. The International Classification of Disease, 9th Revision, Clinical Modification (ICD-9-CM) was used to identify individual health status. This study was approved by the Institutional Review Board of China Medical University.

### Study Subjects

We identified patients with ESRD newly diagnosed in 1998–2009 yearly from the sub-dataset with one million insured people. The patients who had completed the seasonal influenza vaccination (ICD-9-CM V04.7 and V04.8) were designated in the vaccine cohort, with the date of vaccination defined as the index date for measuring the follow-up period. The patients who received pneumococcal vaccine were excluded (N = 75). Newly diagnosed HD patients without both influenza and pneumococcal vaccinations were also identified annually as the non-vaccine cohort. The corresponding non-vaccine cohort was selected randomly from the same month of the index year.

### Outcome Measures

Both cohorts were followed up since the index date for one year or until selected events, death, or withdrawal from the insurance. We were interested in outcome events of total hospitalization, pneumonia or influenza (ICD-9-CM codes 480-487), hospitalization for septicemia, bacteremia, or viremia (ICD-9-CM codes 038.x, 790.7, and 790.8), hospitalization for heart disease (ICD-9-CM codes 401–429), respiratory failure (ICD-9-M codes 518.81, 518.82, 518.83, 518.84, 799.1), or intensive care unit (ICU) admission or death. The history of coronary artery disease (ICD-9-CM codes 410–413, 414.01–414.05, 414.8, and 414.9), congestive heart failure (ICD-9-CM codes 428, 398.91, 402.x1), cancer (ICD-9-CM codes 140–149, 150–159, 160–165, 170–175, 179–189, 190–199, 200, 202, 203, 210–213, 215– 229, 235–239, 654.1, 654.10, 654.11, 654.12, 654.13, 654.14), hyperlipidemia (ICD-9-CM codes 272), hypertension (ICD-9-CM codes 401–405), diabetes (ICD-9-CM codes 250), atrial fibrillation (ICD-9-CM codes 427.31), stroke (ICD-9-CM codes 430–438), chronic hepatitis (ICD-9-CM codes 571, 572.2, 572.3, 572.8, 573.1, 573.2, 573.3, 573.8, 573.9), and chronic obstructive pulmonary disease (ICD-9-CM codes490, 491– 495, 496) were identified as comorbidities before the index date.

### Statistical Analysis

Proportion of annual influenza vaccination in HD patients was measured chronologically from 1998–2009. Demographic characteristics of HD patients and prevalence of comorbidities were compared between the vaccine and non-vaccine cohorts, and tested using Chi-square test for categorical variables and t-test for continuous variables. Incidence rates for hospitalization events, infections (pneumonia/influenza, septicemia, bacteremia and viremia), heart disease, respiratory failure, intensive care unit (ICU) admission, and mortality were estimated for each cohort. The vaccine to non-vaccine cohort incidence rate ratio (IRR) and 95% confidence interval (CI) were estimated using Poisson regression. Cox proportional hazards regression model was also used to estimate the hazard ratio (HR) for each of these events and the corresponding 95% CI, within one year after the vaccination. The Cox proportional hazards regression model were also used to estimate pneumonia/influenza-free rates, controlling for age, sex, hyperlipidemia, hypertension, congestive heart failure, chronic hepatitis, chronic obstructive pulmonary disease and calendar year. Similarly, the probability free of respiratory failure, ICU admission, and mortality were plotted using the Cox proportional hazards regression model. The influenza vaccination changed annually, we used the time-dependent Cox model to further evaluate the mortality risk related to the vaccination for multiple years. SAS version 9.1 (SAS Institute, Cary, NC, USA) was used for data analyses; p <0.05 was considered to indicate statistical significance.

## Results

From 1998 to 2009, new patients receiving HD increased annually from 264 cases in 1998 to a peak of 368 cases in 2007, and then declined to 347 cases in 2009 (Table 1). The proportion of patients receiving influenza vaccine increased from 1.14% to a peak of 38.0% in 2009. A total of 29 patients received both seasonal influenza and influenza A (H1N1) vaccines in 2009. A total of 103 patients received seasonal influenza vaccination only (data not shown). Overall, 831 patients were in the influenza vaccine cohort, which comprised less portion of women than 3187 patients in the non-vaccine comparison cohort (Table 2). The vaccine cohort mainly included the elderly, with the mean age much higher than that of the non-vaccine cohort (70.2± 9.96 vs. 59.4±14.5 years). The vaccine cohort also tended to be more prevalent with comorbidities. There was no patient who received transplantation or was shifted to peritoneal dialysis during the follow-up period.

**Table 1:** Proportion of annual influenza vaccination in hemodialysis patients.

	Influenza vaccine			
Year	N	Yes	No	Proportion (%)
1998	264	3	261	1.14
1999	270	16	254	5.93
2000	313	50	263	15.97
2001	315	48	267	15.24
2002	335	80	255	23.88
2003	361	90	271	24.93
2004	363	78	285	21.49
2005	397	89	308	22.42
2006	355	90	265	25.35
2007	368	70	298	19.02
2008	330	85	245	25.76
2009	347	132	215	38.04

**Table 2:** Demographic status and comorbidity at baseline in hemodialysis patients with and without influenza vaccination.

	Influenza vaccine		p-value
	Yes (n = 831)	No (n = 3187)	
Age, mean ± SD	70.2±9.96	59.4±14.5	<0.0001^#^
Stratify age			
18–39	8(0.96)	284(8.91)	<0.0001
40–64	127(15.3)	1714(53.8)	
65+	696(83.8)	1189(37.3)	
Gender			
Women	413(49.7)	1635(51.3)	0.41
Men	418(50.3)	1552(48.7)	
Comorbidity			
CAD	401(48.3)	997(31.3)	<0.0001
CHF	253(30.5)	672(21.1)	<0.0001
Cancer	82(9.87)	236(7.41)	0.02
Hyperlipidemia	466(56.1)	1342(42.1)	<0.0001
Hypertension	776(93.4)	2795(87.7)	<0.0001
Diabetes	552(66.4)	1694(53.2)	<0.0001
Atrial fibrillation	26(3.13)	67(2.10)	0.08
Chronic hepatitis	323(38.9)	874(27.4)	<0.0001
COPD	356(42.8)	917(28.8)	<0.0001

Abbreviations: CAD, coronary artery disease; CHF, congestive heart failure; COPD; chronic obstructive pulmonary disease

Chi-square test; ^#^: Two-sample t-test

The analysis for age-specific hospitalization shows that most of patients hospitalized were the elderly (≧ 65 years) (Table 3). The overall crude hospitalization rate was 11.0% higher in the vaccine cohort than in the non-vaccine cohort (99.4 vs. 89.4 per 100 person-years). However, the vaccine cohort had an adjusted HR of 0.81(95% CI = 0.72–0.90) after controlling for sex, age, coronary artery disease; congestive heart failure; chronic obstructive pulmonary disease, cancer, stroke, and calendar year. The elderly with vaccination had significant lower rate than those without (100.8 vs. 133.9 per 100 person-years), with an adjusted IRR of 0.73 (95% CI 0.64–0.82).

**Table 3:** Comparison in hospitalization rates between hemodialysis patients with and without influenza vaccination.

	Vaccine			Non-vaccine					
Outcome	Event	PY	Rate^#^	Event	PY	Rate^#^	IRR^*^(95% CI)	Adjusted IRR(95% CI)	Adjusted HR (95% CI)
Total Hospitalization	459	462	99.4	1688	1888	89.4	1.11(0.96, 1.28)	0.80(0.69, 0.94)	0.82(0.73, 0.91)
Age, years									
18–39	1	5	20	113	205	55.1	0.40(0.03, 5.54)	0.36(0.03, 4.76)	0.36(0.05, 2.62)
40–64	59	62	95.2	820	1118	73.3	1.31(0.92, 1.86)	1.22(0.86, 1.74)	1.16(0.89, 1.52)
65+	399	396	100.8	755	564	133.9	0.75(0.64, 0.89)	0.70(0.59, 0.82)	0.73(0.64, 0.82)

Rate^#^, incidence rate, per 100 person-years; IRR^*^, incidence rate ratio;

HR, hazard ratio adjusted for sex, age, coronary artery disease; congestive heart failure; chronic obstructive pulmonary disease, cancer, stroke, and calendar year

Table 4 shows that the incidence of heart disease, the major cause of hospitalization, was higher in the vaccine cohort than in the non-vaccine cohort (65.3 vs. 51.7 per 100 person-years). However, the adjusted HR of the vaccine cohort was 0.85 (95% CI 0.75–0.96). The incidence rates of pneumonia/influenza, hospitalization for septicemia/bacteremia/viremia, and respiratory failure were also higher in the vaccine cohort than in the non-vaccine cohort. However, adjusted HRs also demonstrated protective association from the vaccination. Compared with the patients in the non-vaccine cohort, those in the vaccine cohort were less likely to be admitted in the ICU [2.6 vs. 6.8 per 100 person-years; adjusted HR 0.2 (95% CI 0.12–0.33)] and to be deceased [18.5 vs. 21.0 per 100 person-years; adjusted HR 0.50 (95% CI 0.41–0.60)].

**Table 4:** Hospitalization, intensive care unit utilization, and mortality comparison between hemodialysis patients with and without influenza vaccination.

	Vaccine			Non-vaccine					
Outcome	Event	PY	Rate^#^	Event	PY	Rate^#^	IRR^*^(95% CI)	Adjusted IRR (95% CI)	Adjusted HR (95% CI)
Pneumonia/influenza^†^	139	620	22.4	445	2584	17.2	1.30(1.08, 1.56)	0.77(0.64, 0.93)	0.78(0.64, 0.96)
Septicemia, bacteremia, and viremia^†^	103	637	16.2	347	2661	13.0	1.24(1.02, 1.50)	0.66(0.47, 0.92)	0.72(0.56, 0.88)
Heart disease^†^	341	522	65.3	1138	2203	51.7	1.26(1.08, 1.47)	0.83(0.71, 0.97)	0.85(0.75, 0.96)
Respiratory Failure^†^	66	653	10.1	262	2697	9.7	1.04(0.84, 1.29)	0.52(0.42, 0.64)	0.53(0.41, 0.70)
Intensive care unit admission^†^	17	660	2.6	184	2696	6.8	0.38(0.27, 0.53)	0.19(0.14, 0.27)	0.20(0.12, 0.33)
Mortality^†^	123	665	18.5	581	2765	21.0	0.88(0.73, 1.07)	0.49(0.41, 0.59)	0.50(0.41, 0.60)

Rate^#^, incidence rate, per 100 person-years; IRR^*^, incidence rate ratio

Hospitalization^†^: adjusted for age, CAD, CHF, cancer, COPD, stroke, and calendar year

Hospitalization for pneumonia/influenza^†^: adjusted for age, sex, CAD, CHF, cancer, diabetes, atrial fibrillation, COPD, and calendar year

Hospitalization for septicemia, bacteremia, and viremia^†^: adjusted for age, cancer, diabetes, atrial fibrillation, and calendar year

Hospitalization for heart disease^†^: adjusted for age, CAD, CHF, hypertension, diabetes, atrial fibrillation, chronic hepatitis, and calendar year

Respiratory Failure^†^: adjusted for age, sex, CAD, cancer, CHF, diabetes, hypertension, chronic hepatitis, and calendar year

Intensive care unit^†^: adjusted for age, cancer, hyperlipidemia, hypertension, diabetes, atrial fibrillation, chronic hepatitis, and calendar year

Mortality^†^: adjusted for age, sex, CAD, cancer, CHF, diabetes, hyperlipidemia, hypertension, chronic hepatitis, atrial fibrillation, COPD, and calendar year

The evaluation for only the elderly also showed that those with vaccination were at lower risks of pneumonia/influenza, hospitalization for septicemia/bacteremia/viremia, and heart disease, ICU admission, respiratory failure, and death (data not shown). The time-dependent Cox model revealed that the overall adjusted HR for mortality associated with the vaccination reduced to 0.30 (95% CI 0.26–0.35), compared with non-vaccinated persons (Table 5).

**Table 5:** Time-dependent Cox model estimated hazard ratios and 95% confidence intervals of mortality risk associated with the vaccination.

	Hazard ratio(95% CI)	
	Crude	Adjusted^†^
Vaccination	0.47(0.41, 0.55)	0.30(0.26, 0.35)

Adjusted HRs^†^: adjusted for age, sex, CHF, diabetes, hyperlipidemia, hypertension, chronic hepatitis, and atrial fibrillation

**Figure 1:**
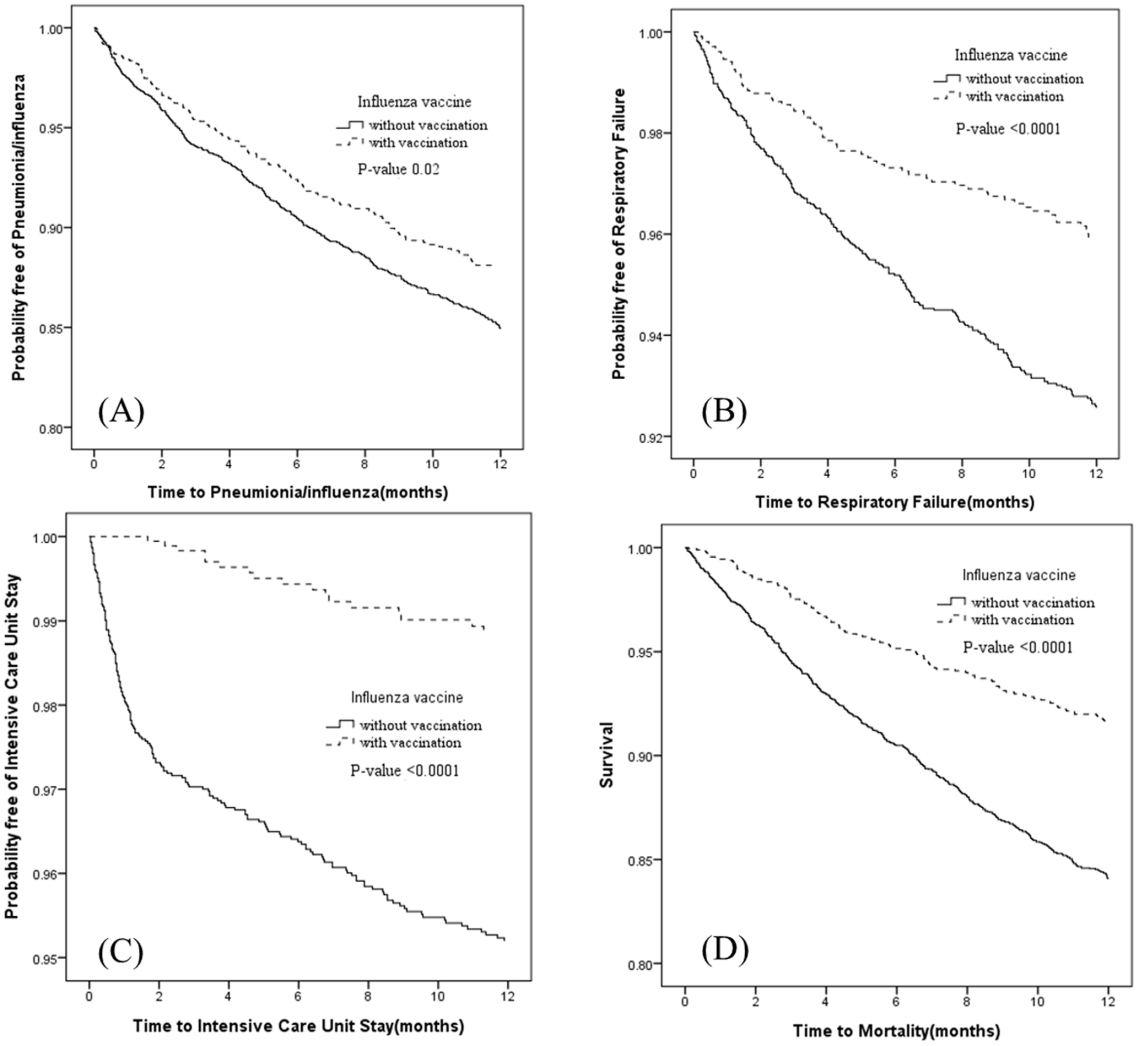
The probability free of pneumonia/influenza, respiratory failure, intensive care unit stay, and mortality for hemodialysis patients with (dashed line) or without (solid line) influenza vaccination.

Figures 1A trough ID show that, compared with the non-vaccine cohort, the vaccine cohort had significantly lower rates of pneumonia/influenza (*P*-value = 0.02) (Figure 1A), respiratory failure (*P*-value <0.0001) (Figure 1B), ICU stay (Figure 1C) (*P*-value <0.0001), and mortality (Figure 1D) (*P*-value <0.0001).

## Discussion

Our study demonstrated that influenza vaccination for ESRD patients receiving HD was associated with lower morbidities, including total hospitalization, ICU admission, pneumonia/influenza, septicemia/bacteremia/viremia, respiratory failure, and heart disease. In addition, influenza vaccination was also associated with lower all-cause mortality risk for 50%. The mortality risk decreased further to have an overall adjusted HR of 0.30 as subjects with vaccinations for multiple years were counted. Consistent with the present study, a previous research in the US reported that the risks of hospitalization and death for HD patients vaccinated against influenza are lower than those of unvaccinated patients, with odds ratios of 0.93 (95% 0.90–0.95) and 0.77 (0.73–0.81), respectively [14]. Moreover, they found that vaccination was significantly associated with lower risks of cause-specific mortality, including cardiac and infectious deaths. Recent reports demonstrated that influenza vaccination was associated with a lower mortality risk [12], [13]. The mortality hazard was reduced further for patients receiving both pneumococcal and influenza vaccinations. On the contrary, McGrath et al. recently reported little clinical benefits of influenza vaccination on preventing hospitalization and death [16]. However, their study did not consider the confounding effect of pneumococcal vaccination. Therefore, our study observed closely the effectiveness of influenza vaccination, by excluding the effect of pneumococcal vaccine. In addition, these studies analyzed prevalent ESRD patients. Length of time with ESRD may have confounding effect on the outcomes.

Compared with healthy control subjects, ESRD patients possess suboptimal immune response rates but develop satisfying protection rates to influenza vaccination [18], [19], [20], [21], [22]. The antibody response rates to influenza vaccination (defined as a four-fold increase in hemagglutination inhibition titers) vary from 7% to 89% [18], [19], [20], [21], [22]. The seroprotection rates (defined as hemagglutination inhibition titers≥40) range from 46% to 93%, depending on the specific strain measured [18], [19], [20], [21], [22]. Booster vaccination fails to improve the immune response [20], [21], [23]. Influenza vaccination is safe without causing major adverse effects in HD patients [20], [23], and the number of minor adverse reactions is low [20].

Since 1998, the Taiwan NHI program has started to offer influenza vaccinations to high-risk subjects, including ESRD patients and the elderly subjects. Majority of the vaccinated subjects receive this service annually between October 1 and December 31. However, the influenza vaccination rate in this study was low for incident HD patients, especially in the beginning years (1998 and 1999). The higher vaccination rate in 2009 was likely due to the pandemic novel influenza A (H1N1) in April 2009 [24]. The low vaccination rate may be attributed to the lack of awareness on its benefit, fear of adverse reactions, and lack of physician recommendation [25]. The goal of the World Health Organization is to increase the annual influenza vaccination rate to 90% by 2010 for ESRD patients.

Influenza may lead to viral pneumonia and bacterial superinfection. HD patients are susceptible to pulmonary infection. Transient hypoxemia occurs during dialysis because of leukocyte migration in the pulmonary vasculature and loss of carbon dioxide [6]. In addition, HD patients are exposed to other patients and medical staff. Our study demonstrated that influenza vaccination was associated with a lower risk of pneumonia/influenza during the first 3 months after vaccination (data not shown). Seroprotection was maintained at least for 3 months and up to 6 months after influenza vaccination in patients with renal diseases [26].

Consistent with the findings of the present study, Gilbertson reported that influenza vaccination is associated with a lower risk of heart disease in ESRD patients [14]. For patients with chronic kidney disease, an association between influenza vaccination and atherosclerotic heart disease event rates also exists [27]. Systemic inflammation caused by influenza infection may lead to endothelial injury, impaired vasodilatation, and enhanced thrombosis formation [28]. Inflammation is highly associated with increasing cardiovascular mortality [29], [30].

The current study has several limitations. First, the NHI database provided limited information on socio-demographic characteristics, with unavailable information on marital status, educational level, smoking habit, body-mass index, and laboratory data, such as hemoglobin, albumin, and residual renal function. These variables cannot be adjusted in the analysis. Furthermore, certain information on chronic conditions, such as hyperlipidemia and hypertension, were not available for some individuals. However, HD patients were visited by health care professionals frequently, the claim data were reliable. Moreover, the decision to receive vaccination may have been affected by socioeconomic status, as well as availability of health care and medical providers. Although multivariate analysis was used, selection bias may occur. Fourth, this study focused only on all-cause mortality because the cause of death cannot be obtained from the database. Finally, the strain and virulence of the predominant virus and the match between the circulating virus strain and the virus strain selected in the vaccine varied from year to year. Therefore, we adjusted the calendar year for temporal effect.

## Conclusions

Our study provides evidence that influenza vaccination is associated with the reduction of mortality and morbidity in HD patients. Thus, HD patients should be vaccinated annually. More large-scale prospective studies are necessary to analyze the issue.
